# Apolipoprotein E-specific innate immune response in astrocytes from targeted replacement mice

**DOI:** 10.1186/1742-2094-3-10

**Published:** 2006-04-07

**Authors:** Izumi Maezawa, Nobuyo Maeda, Thomas J Montine, Kathleen S Montine

**Affiliations:** 1 Department of Pathology, University of Washington, Seattle, WA, USA; 2 Department of Pathology, University of North Carolina, Chapel Hill, NC, USA

## Abstract

**Background:**

Inheritance of the three different alleles of the human apolipoprotein (apo) E gene (*APOE*) are associated with varying risk or clinical outcome from a variety of neurologic diseases. ApoE isoform-specific modulation of several pathogenic processes, in addition to amyloid β metabolism in Alzheimer's disease, have been proposed: one of these is innate immune response by glia. Previously we have shown that primary microglia cultures from targeted replacement (TR) APOE mice have apoE isoform-dependent innate immune activation and paracrine damage to neurons that is greatest with TR by the ε4 allele (TR APOE4) and that derives from p38 mitogen-activated protein kinase (p38MAPK) activity.

**Methods:**

Primary cultures of TR APOE2, TR APOE3 and TR APOE4 astrocytes were stimulated with lipopolysaccharide (LPS). ApoE secretion, cytokine production, and nuclear factor-kappa B (NF-κB) subunit activity were measured and compared.

**Results:**

Here we showed that activation of primary astrocytes from TR APOE mice with LPS led to TR APOE-dependent differences in cytokine secretion that were greatest in TR APOE2 and that were associated with differences in NF-κB subunit activity.

**Conclusion:**

Our results suggest that LPS activation of innate immune response in TR APOE glia results in opposing outcomes from microglia and astrocytes as a result of TR APOE-dependent activation of p38MAPK or NF-κB signaling in these two cell types.

## Background

Humans are different from other mammals in that we have 3 common alleles of the apolipoprotein E gene (*APOE*): the ε2 (*APOE2*), ε3 (*APOE3*), and ε4 (*APOE4*) alleles [1]. Numerous genetic studies have associated inheritance of *APOE4 *with increased risk, earlier onset, or poorer clinical outcome for a number of neurodegenerative diseases, including Alzheimer's disease (AD), Parkinson's disease (PD), amyotrophic lateral sclerosis (ALS), traumatic brain injury, and HIV-encephalitis [2-10]. At least for AD, inheritance of *APOE2 *is associated with apparent neuroprotection, perhaps related to delayed onset of illness by many years [11]. While apoE isoforms play a role in the metabolism of beta amyloid (Aβ) peptides and thereby may modulate the risk of developing AD [12], the influence of inheriting different *APOE *alleles extends well beyond diseases thought to involve Aβ peptide-mediated neurotoxicity, as noted above. For this reason, other apoE-isoform specific mechanisms likely exist to explain the apparent influence of *APOE *alleles on such a broad spectrum of neurologic diseases; indeed, several have been proposed including synaptic stabilization, biologically active proteolytic fragments of apoE, anti-oxidant activity, and nitric oxide (NO) production [13-16]. ApoE also has an immune modulatory function, at least in the peripheral adaptive immune response to some bacteria and viruses [17]. We have recently shown that microglia from mice with targeted replacement (TR) of the mouse apoE gene with the coding sequences of human APOE alleles activated with LPS display an apoE isoform-specific innate immune response and result in apoE isoform-specific paracrine damage to neurons, both of which are dependent on p38 mitogen-activated protein kinase (p38MAPK) -mediated signaling.

One commonly used approach to investigate selectively innate immune response in neurodegeneration is to use a specific stimulus, lipopolysaccharide (LPS) [18-25]. LPS specifically activates CD14/Toll-like receptor (TLR) 4 co-receptors with subsequent increased gene transcription mediated through a bifurcated pathway that is dependent on both nuclear factor-kappa B (NF-κB) and p38MAPK signaling [26,27]. Indeed, LPS activation of CD14/TLR4 co-receptors on microglia leads to indirect damage to neurons and oligodendroglia in culture and *in vivo *[22,28-30]. Moreover, a role for CD14/TLR4 co-receptors is now understood to extend well beyond endotoxemia, as they are important in innate immune response to several endogenous ligands [31]. Indeed, CD14 binds Aβ fibrils and is responsible for most of Aβ–stimulated microglial-mediated neurotoxicity [32]. In addition, peptides and neoantigens expressed by apoptotic cells also activate this pathway [33]. Here we tested the hypothesis that innate immune response from CD14/TLR4 activation would show isoform-specific differences in primary cultures of astrocytes from TR APOE mice.

## Methods

### Materials

Cell culture solutions and supplies were from GIBCO (Grand Island, NY). Poly-ornithine (0.01%) was from Sigma (St. Louis, MO). 4–15% SDS-polyacrylamide gels were from BioRad (Hercules, CA). LPS and the NO assay kit were from Calbiochem (La Jolla, CA). Primary antibodies used were polyclonal anti-human apoE antibody from Dako Corporation (Carpinteria, CA) and polyclonal anti-glial fibrillary astrocytic protein (GFAP) antibody from Novus Biologicals (Littleton, CO). The NF-κB transcription factor assay kit and purified human HDL were from Chemicon International (Temecula, CA)

### Mice

Homozygous APOE2, APOE3 and APOE4 targeted replacement (TR) mice 'humanized' at *apoE *were developed by Dr. Maeda and colleagues [34,35]. Briefly, human *APOE *genomic fragments were used to replace mouse *apoE *via homologous recombination. All three lines of TR APOE mice contain chimeric genes consisting of mouse 5' regulatory sequences continuous with mouse exon 1 (noncoding) followed by human exons (and introns) 2–4 [34]. These mice were backcrossed greater than six generations to C67BL/6 genetic background. Mice were housed in an ALAC-approved vivarium and methods approved by a University of Washington International Use and Care of Animals (IACUC) Committee.

### Astrocyte cultures

Primary cultures of 1-day-old mouse cerebral cortical astrocytes were prepared according to the method of Gebicke-Haerter, *et al*. [36]. Confluent cultures were used on the 7^th ^day *in vitro *(DIV). Our preparations were ≥ 93% pure for astrocytes, as demonstrated by glial fibrillary acidic protein (GFAP) antibody. Astrocytes were exposed to LPS in serum-free medium at a final concentration of 100 ng/ml (20 ng/10^5 ^cells). Vehicle control for LPS exposure was PBS.

### Western blot analysis

Conditioned (serum-free) medium was removed from astrocyte cultures following LPS or vehicle exposure and centrifuged at 13,000 × *g *for 2 min at 4°C to remove cell debris. Equal volumes of conditioned media were diluted with 6X sample buffer (0.35 M Tris, 30% glycerol, 10% SDS, 0.93 g DTT, 1.2 mg bromophenol blue), heated to 95°C for 5 min, subjected to SDS PAGE, transferred to PVDF membranes, and analyzed and quantified as previously described [37]. Anti-human apoE (Dako) was used at 1:2000 dilution. Secondary antibody was HRP-conjugated anti-rabbit (1:3000).

### NO detection

NO levels in conditioned media following incubation with LPS or vehicle were measured using a colorimetric NO assay kit (Calbiochem) where nitrate is first converted to nitrite by the NADH-dependent nitrate reductase, followed by nitrite measurement using the Griess Reagent.

### Cytokine measurements

Conditioned medium following incubation with LPS or vehicle was screened for cytokines with an array method, and selected cytokines further quantified individually by sandwich ELISAs. The bead-based Liquichip™ Mouse 10-Cytokine Kit (Qiagen Inc, Valencia CA) was used to simultaneously screen conditioned media for the following cytokines: GM-CSF, interferon (INF)-γ, interleukin (IL)-1β, -2, -4, -5, -6, -10, -12, and tumor necrosis factor (TNF) –α. This kit uses cytokine antibodies immobilized on LiquiChip™ beads with distinct bead codes, which are added to conditioned media samples. Bead-bound cytokines are detected using a mixture of biotinylated cytokine-specific monoclonal antibodies and Streptavidin-PE. The specific bead code assigned to each of the 10 cytokines enables their unambiguous identification and quantification by a Luminex 100 X-Map reader using Qiagen software. Next, IL-6, IL-1β, and TNF-α from conditioned media were separately quantified by sandwich ELISAs using DuoSet ELISA development kits for each cytokine (R&D Systems, Minneapolis, MN).

### NF-κB activity

NF-κB activity following incubation with LPS or vehicle was measured using an NF-κB transcription factor assay kit from Chemicon International. Briefly, cells were rinsed with PBS, lysed in Buffer A (10 mM HEPES (pH7.9), 1.5 mM MgCl_2_, 10 mM KCl, 0.5 mM DTT, 0.1% Triton X-100 and protease inhibitor cocktail), and a nuclear extract prepared in Buffer B (20 mM HEPES (pH 7.9), 1.5 mM MgCl_2_, 0.42 M NaCl, 0.2 mM EDTA. 0.5 mM DTT, 1.0% Igepal CA-630, 25% (v/v) glycerol, and protease inhibitor cocktail). Double-stranded biotinylated oligonucleotide containing the flanked consensus sequence for NF-κB was mixed with the nuclear extract and the mixture immobilized on a streptavidin-coated chemiluminescent plate, followed by immunologic detection of the bound NF-κB transcription factor subunits p50 and 065.

## Results

We have recently reported that LPS activation of TR APOE glial-wt neuron mixed cultures for 24 hours results in apoE isoform-specific paracrine damage to neurons [30]. For activated microglia, TR APOE4 is more neurotoxic than TR APOE2 or APOE3. For activated astrocytes, which produce much less neurotoxicity than microglia, both TR APOE4 and TR APOE3 are mildly damaging to neurons, while TR APO2 shows no neurotoxic effect. In this previous work, we pursued apoE-isoform specific mechanisms in LPS-activated microglia and showed that these were p38MAPK-dependent. Here, we pursued the basis of apoE isoforms-specific differences in LPS activation of astrocytes from these TR mice.

**Figure 1:**
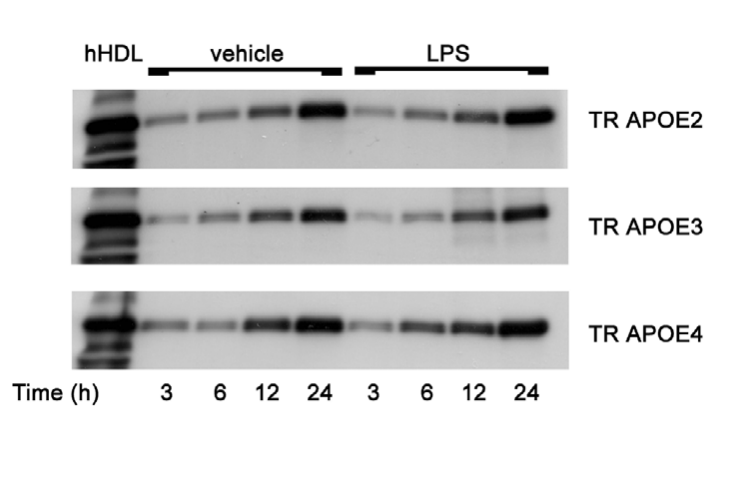
**ApoE secretion following LPS stimulation. **Mouse cerebral primary astrocyte cultures were incubated in serum-free medium with 100 ng/ml LPS or vehicle (PBS) for 24 hours. 20 μl of conditioned medium was collected at 3, 6, 12, and 24 hrs after exposure, spun briefly, mixed with 4 μl of 6X sample buffer (0.35 M Tris, 30% glycerol, 10% SDS, 0.93 g DTT, and 1.2 mg bromophenol blue), and relative concentrations of apoE determined by Western blotting. Human high-density lipoprotein (hHDL) prepared in the same sample buffer was included as a positive control.

We first showed that there was no difference among the three TR APOE astrocytes in the amount of secreted apoE following LPS exposure for up to 24 hours (Figure 1), in agreement with our findings for microglia [30]. We also determined that similar to microglia, there was no difference in medium nitrate plus nitrite levels (a measure of NO secretion) compared to wild type (wt) at 12 or 24 hours after LPS exposure (P > 0.05), although we did observe increased medium nitrate plus nitrite levels in TR APOE4 (205 ± 41 % of wt) but not TR APOE2 (116 + 15% of wt) astrocytes 72 hours after LPS incubation. As with microglia, this temporal mismatch suggests increased NO secretion by TR APOE4 lies distal to the processes underlying the TR-APOE isoform-specific differences in astrocyte-mediated neurotoxicity seen within 24 hours of LPS incubation [30].

**Figure 2:**
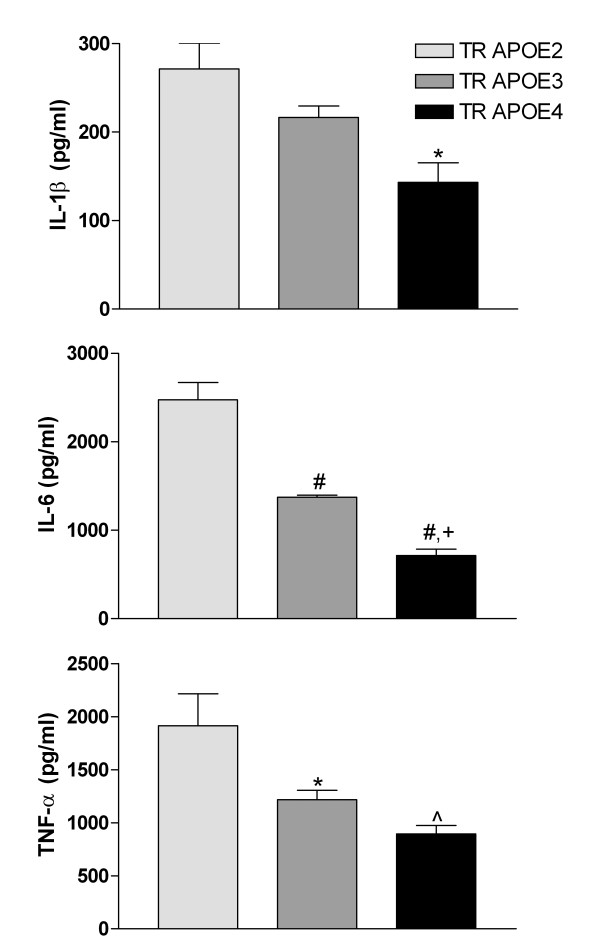
**Cytokine secretion following LPS stimulation. **Mouse cerebral primary astrocyte cultures were incubated in serum-free medium with 100 ng/ml LPS or PBS for 12 hr, medium collected, and IL-1β, IL-6 and TNF-α concentrations determined by ELISA. All cytokines were below the limit of detection in PBS-exposed cultures. Data are mean ± SEM (n = 4 to 8 separate cultures per group). One-way ANOVA showed P < 0.05 for all three cytokines. *P < 0.05, ^^^P < 0.01, or ^#^P < 0.001 for Bonferroni-corrected posttests for LPS-incubated TR APOE3 or TR APOE4 *vs*. TR APOE2; ^+^P < 0.01 for TR APOE4 *vs*. TR APOE3.

Previously, we observed TR APOE-dependent differences in cytokine secretion by microglia in response to LPS exposure [30]. Here we measured cytokine secretion in response to LPS in the three TR APOE astrocyte cultures. We screened for changes in medium cytokine concentrations using the LiquiChip™ Mouse 10-Cytokine assay and a Luminex 100 X-Map reader that simultaneously determines 10 mouse cytokines in medium from TR APOE astrocytes. The cytokines quantified were GM-CSF, INF-γ, IL-1β, IL-2, IL-4, IL-5, IL-6, IL-10, IL-12, and TNF-α. Only IL-6 and TNF-α changed significantly following LPS exposure for 12 hr; IL-1β was near the limit of detection for this assay. The magnitude of induction for these cytokines was TR APOE-dependent with IL-6 and TNF-α concentrations following the gradient of TR APOE2 > TR APOE3 > TR APOE4. We confirmed our IL-6 and TNF-α findings with individual ELISAs and extended our analysis to IL-1β, since many others have shown it to be overexpressed and secreted from LPS-stimulated glia; TR APOE-dependence of IL-1β secretion followed the same pattern as the other two cytokines (Figure 2).

**Figure 3:**
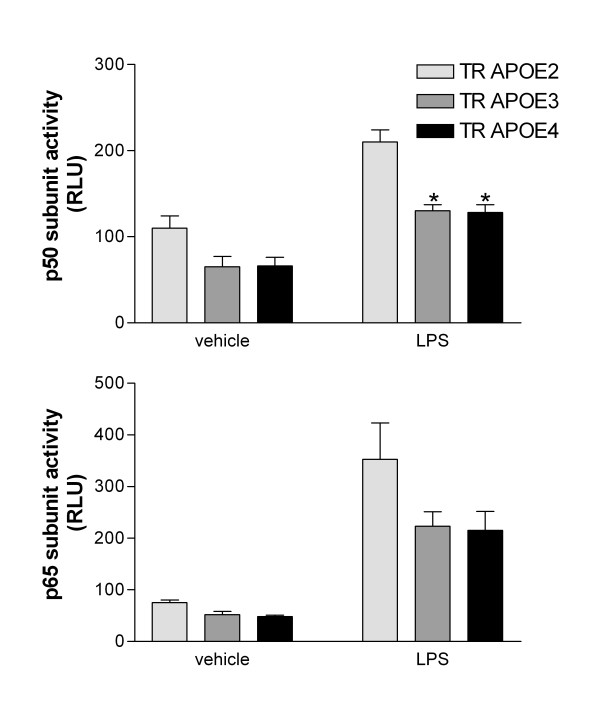
**NF-κB activity following LPS stimulation**. NF-κB p50 and p65 subunit activity was determined in nuclear extracts from mouse cerebral primary astrocyte cultures exposed to vehicle (PBS) or LPS for 12 hr using an NF-κB transcription factor assay kit (Chemicon). Data are expressed as average relative light units (RLU) ± SEM (n = 4 for each group). Two-way ANOVA for p50 data had P < 0.0001 for TR APOE and vehicle *vs*. LPS, but P > 0.05 for interaction between these terms. Two-way ANOVA for p60 data had P < 0.05 for TR APOE and P < 0.0001 for vehicle *vs*. LPS, but P > 0.05 for interaction between these terms. *P < 0.01 for Bonferroni-corrected posttests for LPS-exposed TR APOE3 or TR APOE4 *vs*. TR APOE2.

LPS activation of CD14/TLR4 co-receptors leads to subsequent increased gene transcription mediated through a bifurcated pathway that is dependent on NF-κB and p38MAPK signaling. We have previously demonstrated apoE isoform-specific p38MAPK activation following LPS exposure of microglia but not astrocytes [30]. We therefore determined the activity of two NF-κB subunits, p50 and p65, in astrocytes from TR APOE mice (Figure 3). Following LPS exposure, both p50 and p65 activity significantly increased in all 3 genotypes. p50 activity showed an apoE isoform-specific increase, with a larger increase in TR APOE2 than the other two (P < 0.01 for both) and no difference between TR APOE3 and TR APOE4. p65 showed a similar trend in apoE isoform-specific effect; however, this was not significantly different in corrected multiple comparison tests.

## Discussion

Inheritance of *APOE *alleles is associated with varying clinical outcomes in several neurodegenerative diseases, including AD, PD, ALS, head trauma, multiple sclerosis, and HIV-encephalitis [2-10]. Although apoE isoforms likely modulate AD pathogenesis by influencing metabolism of Aβ [12,38], the pathophysiologic significance of apoE isoforms appears to go beyond interacting with Aβ since these other diseases of brain are not thought to involve Aβ peptides in their pathogenesis. Indeed, others have suggested more general mechanisms of neurotrophism or neurotoxicity from inheritance of different *APOE *alleles that potentially could contribute to multiple neurologic diseases [13-16]. Since activation of innate immunity also is associated with these same diseases, we tested the hypothesis that apoE isoforms may act by modulating glial innate immune response and thereby altering neurotoxicity. Previously, we showed that microglia from TR APOE mice show apoE isoform-specific innate immune activation and paracrine damage to neurons that was greatest with TR APOE4 and dependent on p38MAPK signaling [30]. Here, we showed that identical activation of astrocytes from these same TR APOE mice had apoE isoforms-specific innate immune response that was greatest with TR APOE2 astrocytes and associated with NF-kB-mediated signaling.

We used a model of selective activation of CD14/TLR4 co-receptors that is now appreciated to initiate innate immune response to endogenous ligands relevant to neurodegenerative diseases such as Aβ fibrils as well as peptides and neoantigens expressed by apoptotic cells [32,33]. LPS activation of CD14/TLR4 co-receptors leads to increased gene transcription through a bifurcated pathway; one arm is NF-κB-dependent and the other is p38MAPK-dependent [26,27]. Our data indicated that the intracellular signaling that mediates altered gene transcription in response to LPS is different between astrocytes and microglia expressing TR APOE. Specifically, NF-κB-mediated signaling, which is associated with immune modulation and protection of cells from undergoing apoptosis [39], was greatest in TR APOE2 astrocytes, the only cell line that did not yield paracrine damage to neurons following activation with LPS [30]. In contrast, apoE isoforms-specific effects in microglia, including much more extensive paracrine damage to neurons, was associated with p38MAPK signaling [30]. We speculate that the inverse relationship between low-level neurotoxicity associated with LPS-activated astrocytes that we reported previously [30] and innate immune activation may be related to diminished NF-κB-dependent trophic factors in TR APOE3 and TR APOE4 astrocytes.

## Conclusion

Our results suggest that LPS activation of innate immune response in TR APOE glia results in opposing outcomes from microglia and astrocytes as a result of TR APOE-dependent activation of p38MAPK or NF-κB signaling in these two cell types.

## Abbreviations

AD (Alzheimer's disease); ALS (amyotrophic lateral sclerosis); apo (apolipoprotein); *APOE *(human apoE gene); Aβ (beta amyloid); DIV (days *in vitro*); GFAP (glial fibrillary astrocytic protein); hHDL (human high density lipoprotein); IL (interleukin); INF (interferon); LPS (lipopolysaccharide); NF-κB (nuclear factor kappa B); NO (nitric oxide); p38MAPK (p38 mitogen-activated protein kinase); PD (Parkinson's disease); RLU (relative light units); TLR (Toll-like receptor); TNF (tumor necrosis factor); TR (targeted replacement); wt (wild type).

## Competing interests

The author(s) declare that they have no competing interests.

## Authors' contributions

IM carried out the experiments described. NM developed the mouse line that was used in all experiments. TJM conceived the study and its design and helped to draft the manuscript. KSM assisted in experimental design, analyzed the data, and drafted the manuscript.
